# aFGF Targeted Mediated by Novel Nanoparticles-Microbubble Complex Combined With Ultrasound-Targeted Microbubble Destruction attenuates Doxorubicin-Induced Heart Failure via Anti-Apoptosis and Promoting Cardiac Angiogenesis

**DOI:** 10.3389/fphar.2021.607785

**Published:** 2021-04-27

**Authors:** Nan-Qian Zhou, Zhi-Xin Fang, Ning Huang, Yue Zuo, Yue Qiu, Li-Juan Guo, Ping Song, Jian Xu, Guang-rui Wan, Xin-Qiao Tian, Ya-ling Yin, Peng Li

**Affiliations:** ^1^ Department of Ultrasonography, Henan Provincial People’s Hospital, People’s Hospital of Zhengzhou University, Zhengzhou, China; ^2^ Henan International Joint Laboratory of Cardiovascular Remodeling and Drug Intervention, Xinxiang, China; ^3^.Xinxiang Key Laboratory of Vascular Remodeling Intervention and Molecular Targeted Therapy Drug Development, Xinxiang, China; ^4^ College of Pharmacy, Xinxiang Medical University, Xinxiang, China; ^5^ School of Basic Medical Sciences, Xinxiang Medical University, Xinxiang, China; ^6^ Department of Oncology, First Affiliated Hospital of Xinxiang Medical University, Xinxiang, China

**Keywords:** doxorubicin, heart failure, aFGF-NP, anti-apoptotic, angiogenesis, UTMD

## Abstract

The purpose of this study was to evaluate the protective effect of acidic fibroblast growth factor targeted mediated by novel nanoparticles–cationic lipid microbubbles complex (aFGF–NP + CPMBs) combined with ultrasound targeted microbubble destruction (UTMD)on doxorubicin–induced heart failure (HF)and its mechanism. Heart failure rats induced by intraperitoneal injection with doxorubicin (DOX) to achieve cummulative dose of 15mg/kg for continuous 6 weeks showed left ventricular dysfunction, seriously oxidative stress, cardiomyocyte apoptosis, and decrease of myocardial vascular density. In contrast, aFGF–NP + CPMBs combined with UTMD therapy (3ug/kg, caudal vein injection, twice a week, 6weeks)prominently ameliorated left ventricular dysfunction by increased ejection fraction (EF) and fractional shortening (FS), decreased brain natriuretic peptide (BNP); strengthened the ability of antioxidant stress confirmed by increasing the activity of SOD and reducing the production of MDA; exerted the effect of anti–cardiomyocyte apoptosis and promotion angiogenesis by inhibited Bax expression and increased Bcl–2 expression and platelet endothelial cell adhesion molecule (CD31) expression. Taken together, the research suggested that aFGF targeted mediated by novel nanoparticles–cationic lipid microbubbles complex combined with UTMD should be a promising targeted treatment for heart failure.

## Introduction

 In recent years, ultrasound targeted microbubble destruction (UTMD)as a new non–invasive drug/gene–targeted delivery technology, (Tian et al., 2017) has attracted more and more attention. Under the action of diagnostic or therapeutic ultrasound, the cavitation effect and mechanical effect of ultrasonic microbubble blasting cause temporary open pores on the local endothelial cell membrane and capillaries, through which drugs/genes can be diffused into the tissue to achieve targeted drug or gene delivery and enhances the uptake rate of tissue and therapeutic effect of the drugs. In addition, it can also collect real–time images and data information of specific tissue structure and function changes. As a therapeutic strategy with the advantages of non-invasive, radiation–free,low immunogenicity, low toxicity, (Wang et al., 2020) tissue specificity and controllability, (Sun et al., 2020).

UTMD has been proved to be a promising method for targeting drug/gene transmission and treatment of many diseases, including diagnosis and treatment of malignant tumor, (Sun et al., 2020) Parkinson, (Lin et al., 2020) brain ischaemic, diabetic cardiomyopathy, (Zheng et al., 2019) atherosclerosis, (Yang et al., 2019) transplantation rejection (Liu et al., 2019a).

Heart failure refers to the circulatory dysfunction characterized by the decrease of cardiac output and cannot meet the metabolic needs of the body due to the decrease of myocardial diastolic and systolic function or the limitation of ventricular filling caused by various causes. Heart failure is a social and public health problem that seriously threatens life and health in the process of aging in developed and developing countries all over the world (Li et al., 2019). At present, these drugs for the treatment of heart failure are widely used in the clinic, such as cardiotonic glycosides, diuretics, B–receptor blockers, calcium channel blockers, angiotensin-Ⅱconverting enzyme inhibitors. Although great progress has been made in relieving the symptoms of heart failure, the fatality rate of heart failure is still very high, so there is still an urgent need to develop innovative treatment strategies for increasing the therapeutic effect of heart failure.

Acidic fibroblast growth factor (aFGF)is one of 23 members of the fibroblast growth factor (FGFs) family. The physiological form of aFGF is a polypeptide with a molecular weight of 16 kDa and 155 amino acids. The **a**FGF has important biological functions in human tissues. It binds to fibroblast growth factor receptor (FGFR1-4)in the (RTK)family of transmembrane receptor tyrosine kinases, initiates a variety of intracellular signal transduction pathways, and participates in the promotion of embryogenesis, morphogenesis, angiogenesis, (Cheng et al., 2011) tissue regeneration, (DePaul et al., 2017) nerve injury repair, (Liu et al., 2019b) wound healing and other processes (Wu et al., 2016). It has a broad application prospect in medicine. Therefore, aFGF has a huge therapeutic potentiality for HF. However, aFGF has a short half–life, instability, lack of efficient, safe and controllable myocardial targeted delivery carriers, which limits its clinical application.

Nanoparticles (NP) are non–viral drug and gene delivery carriers. The particle sizes of nanoparticles are mostly between 10 and 1,000 nm. Biodegradable nanoparticles can protect proteins and genes from damage caused by internal and external factors. Because of their small size, nanoparticles can penetrate intact capillaries and endothelial spaces, and can be absorbed by most cells. Nanoparticles have the advantages of high drug loading, high stability *in vivo* and sustained–release drugs, but it is difficult to accumulate in high concentration *in vivo* to achieve efficient targeted therapy. Therefore, an efficient and safe drug delivery system can be constructed by combining UTMD with NP (Tian et al., 2017; Zheng et al., 2019).

The purpose of this research was to determine whether aFGF–NP + CPMBs combined with UTMD was effective in the antagonism of doxorubicin–induced heart failure in animal models. The characteristics of aFGF–NP and CPMBs were detected. In order to further understand the therapeutic effect of aFGF–NP + CPMBs combined with UTMD technology, conventional echocardiography, real–time myocardial contrast echocardiography, histopathology and molecular biology methods were used to evaluate the combination treatment *in vivo* for 6 weeks on improvement in the cardiac structure and functional damages.

## Materials and Methods

### Fabrication and Characterization of aFGF–NP and Cationic Lipid Microbubbles

#### Fabrication of aFGF–NP

 The aFGF–NP was prepared by water-in-water emulsification combined with freeze–drying technique. High concentration aFGF (10 mg/ml, PeproTech, United States) was dissolved in 20% heparin–Poloxamer 188 (HP) solution, stirred and mixed evenly. The mixed solution was then added to 2% gelatin aqueous solution at a volume ratio of 1:2 and dispersed by ultrasound (110 W–15 s). The mixed solution was treated in an ice bath at 5°C, crosslinked and solidified with a prescription amount of D,L-glyceraldehyde, stirred for 5 h, and then freeze–dried to obtain water-in-water carrying aFGF heparinized nanoparticles.

#### Fabrication of Cationic Lipid Microbubbles

 Cationic lipid microbubbles were prepared by ultrasonic freeze–drying. The 1,2-dioleoenoxy-3-trimethyl, aminopropane, distearyl phosphatidylcholine (DSPC) and 1,2-distearoyl-sn-glycero-3-phosphoethanolamine-N-[methoxy (polyethylene glycol)-2000]. (DSPE-PEG2000) were dissolved in a mixture of chloroform and methanol in a molar ratio of 4:5:1, and then was operated by a JY 92-II ultrasonic equipment at 30°C for 3 min (frequency and power parameters:40 kHz, 160 W). After the cationic lipid microbubbles solution was frozen at −20°C in the refrigerator and was lyophilized in vacuum freeze dryer at pressure of 15 Pa (at −48°C for 15 h, and then up to 10°C for no more than 5 h), the cationic lipid microbubbles (150 mg) were added to the mixture of 0.9% normal saline and glycerol (volume ratio = 4:1.5 ml) for dispersion (Zhang et al., 2020). The liquid was transferred to a borosilicate glass bottle and filled with inert gas SF6. Before the cationic lipid microbubbles and drug were mixed, a silver–mercury blender should be used to oscillate the liquid (4,500 r/min, 45 s) in order to produce microbubbles.

#### Fabrication of aFGF Nanoparticles-Cationic Lipid Microbubble Complex

(aFGF−NP+CPMBs)

 The freeze–dried aFGF nanoparticles were re–suspended by PBS, and the concentration was adjusted to 1 mg/ml. AFGF–nanoparticles and the prepared cationic lipid microbubbles were mixed together at a volume ratio of 1:1, and then the mixture was placed at room temperature for 30 min. After 200 g centrifugation for 1 min, the upper suspension layer was taken and re–suspended with 1 ml PBS.

#### Performance Evaluation of Carrying aFGF–Nanoparticles and Cationic Lipid Microbubbles

 The morphological characterization of aFGF-NP was detected by scanning electron microscope, the binding of aFGF-NP and CPMBs was observed by microscope. The particle size and zeta potential of aFGF-NP were measured by dynamic light scattering (DLS). The entrapment efficiency of aFGF-NP was evaluated: about 1.5 ml of aFGF-NP dispersible solution was packed in centrifuge tubes and centrifuged at a speed of 10,000 g for 40 min, then the supernatant was separated, the sample was diluted according to the procedure of ELISA kit (bsk11136, Bioss, China) instruction, and the content detection of aFGF was executed by enzyme-labeled instrument (SpectraMax M3, Molecular Devices Corporation, United States). The entrapment efficiency of aFGF-NP was calculated three times according to the following formula.

$$Entrapment\;efficiency\;\left(EN\%\right)=\frac{\left(total\;amount\;of\;drug\;in\;nanoparticles\;or\;liposome\;suspensions-amount\;of\;free\;drug\right)}{total\;amount\;of\;drug\;in\;nanoparticles\;or\;liposome\;suspensions}\times 100\%$$

#### Animal Experimental Design

 Ninety male SD rats (180–200 g, 45–50 days) were supplied from Experimental Animal Center of Zhengzhou University (Henan Experimental Animal Center). The rats were raised for one week before the beginning of the experimental protocol. All rats were kept in cages with independent ventilation, with a light cycle of 12 h, a temperature of (22 ± 1)°C, and a humidity of (50 ± 10)%, were allowed freely access to food and water. All experimental schemes and treatment were approved by the Ethics Committee of Xinxiang Medical University (Xinxiang, China). The animal model of heart failure was produced by intraperitoneal injection of doxorubicin (2.5 mg/kg according to body weight, once a week for 6 weeks, with a cumulative dose of 15 mg/kg, Duly Biotech Co., Ltd.., Nanjing, China) (Tian et al., 2017).

All rats were randomly divided into six groups (*n* = 15 in each group): The normal group was injected with saline. The animals treated with doxorubicin for 2 weeks (except model group) were followed by tail vein injection of aFGF or aFGF-NP or aFGF-NP + CPMBs combined with UTMD, twice a week for 6 weeks. The treatment method of animals is displayed in Table 1.

**TABLE 1:** The grouping treatment of animal.

	Doxorubicin	aFGF-NP	aFGF	CPMBs	UTMD
GroupA	−	−	−	−	−
GroupB	+	−	−	−	−
GroupC	+	+	−	+	+
GroupD	+	+	−	−	−
GroupE	+	−	+	−	−
GroupF	+	−	+	+	+

Group A (Control group): 1 ml 0.9% Nacl; Group B (DOX–HF group): doxorubicin dissolved in 0.9% Nacl for l ml; Group C (aFGF-NP + UTMD group): a complex of aFGF-NP(3 ug/kg) and 1 ml CPMBs suspension integrated with UTMD; Group D (aFGF-NP group): aFGF-NP(3 ug/kg) dissolved in 0.9% Nacl for l ml; Group E (aFGF group): aFGF (3 ug/kg) dissolved in 0.9% Nacl for l ml; Group F (aFGF + UTMD group): a complex of aFGF (3 ug/kg) and 1 ml CPMBs suspension integrated with UTMD.

#### Targeted Blasting Cationic Lipid Microbubbles and Detected Cardiac Function by Ultrasound

 The rats were anesthetized with 3% chloral hydrate (1 ml/100 g i. p) and were tied in the supine position. Shaved off the chest after using depilation cream and disinfected chest skin with 75% alcohol. The UTMD was executed with the VINNO70 color ultrasonic diagnostic instrument (Feiyinuo Technology Co., Ltd., Suzhou, China)of the X4–12L high–frequency linear array probe with a frequency range of 4–12 MHz. The instrument had the functions of contrast–enhanced ultrasound (CBI)and microbubble cavitation control (VFLASH) and had the weakly focused ultrasonic emission function of adaptive variable focus (ROI). The probe was placed in the left ventricular position of the rat, the coupling agent was filled between the probe and skin. The ultrasound instrument was converted into contrast–enhanced ultrasound mode. The short–axis section of the left ventricle was taken with a focus depth of 3.5–4.0 cm. AFGF–NP or aFGF and CPMBs were slowly injected into the tail vein of rats. When a large amount of CPMBs were seen to enter the myocardial tissue through blood circulation, the VFLASH function of the instrument was turned on, and the whole heart was irradiated in ROI(mechanical index MI = 1.0, pulse length was 26 cycles, VFLASH frequency was 4 MHz, pulse repetition rate was 20 Hz, pulse time was 1.2 s, pulse interval was 2 s), which facilitated more microbubbles to enter the myocardial tissue until the microbubbles disappeared completely in the heart. After 6 weeks treatment schemes and before sacrificing rats, cardiac function was assessed by M-mode echocardiography as described previously (Tian et al., 2017; Zheng et al., 2019).Left ventricular ejection fraction (EF) and fractional shortening (FS), left ventricular internal diameter-systole (LVIDs) and left ventricular internal diameter–diastole (LVIDd) were evenly calculated in three consecutive cardiac cycles.

#### Detection of Biochemical Indexes

 After the echocardiography of the animal hearts were finished, the blood was collected from the abdominal aorta with a blood collection needle. The blood samples were stationary for 30 min, centrifuged at 3,000 rpm, 4°C, for 10 min. Brain natriuretic peptide (BNP) in serum, malondialdehyde (MDA) and superoxide dismutase (SOD) in myocardial tissue homogenate were detected. BNP detection kit (NO: H166), SOD detection kit (NO: A001-1–2) and MDA detection kit (NO: A003-1–1) were purchased from Nanjing Jiancheng Biological Engineering Research Institute Co., Ltd. (Nanjing, China).

#### Histopathologic Analysis

 The heart tissue samples were fixed in 4% paraformaldehyde for 24 h, dehydrated with a series of concentrations of ethanol and tetrahydrofuran, and finally embedded in paraffin. The paraffin–embedded tissue mass was cut into 5 μm slices and stained with Hematoxylin Eosin, Modified Masson's Trichrome Stain (NO: G1345, solarbio, Beijing, China)and Glycogen Periodic Acid Schiff (PAS/Hematoxylin) Stain, which was used to observe the pathological changes of heart tissue. The quantification of fibrosis and glycogen distribution in myocardial tissue was evaluated using a microscope–connected digital camera (Olympus, Nikon, Tokyo, Japan).

#### Terminal Deoxynucleotidyl Transferase–Mediated dUTP Nick–End Labeling

 Myocardial tissue sections with a thickness of 5 μm were stained with TUNEL to determine the double or single–strand breaks of DNA. All paraffin sections were dewaxed with xylene and then treated with gradient ethanol and water. The slices were added with 20 μg/ml DNase–free protease K and incubated at 37°C for 20 min. The slices were washed three times by PBS in order to remove protease K. The samples were added with 50 μL TUNEL detection solution, incubated at 37°C for 60 min and washed again by PBS for three times. The tablets were sealed with anti–fluorescence quenching solution and observed under a fluorescence microscope (Olympus, Tokyo, Japan). The above operations were carried out in accordance with one-step TUNEL cell apoptosis detection kit (C1088, Beyotime, China). The number of apoptotic cardiomyocytes in each slice was counted and averaged from each group.

#### Immunohistochemical Staining

 Put the heart tissue paraffin section slices in a box containing 0.01 M sodium citrate repair solution (PH6.0) and repaired them with boiling heat above 95°C for 20 min 3% H_2_O_2_ was used to block endogenous peroxidase for 30 min and 3%BSA sealed for 30 min at room temperature. The slices were incubated with mouse anti-CD31 (GB12063, 1:300 dilution, Servicebio, China) primary antibody overnight at 4°C independently and each slice was incubated with 70 ul HRP conjugated secondary antibody working solution at room temperature for 30 min. After that, the color can be developed with DAB chromogenic kit and hematoxylin. The positive expression of CD31 were evaluated with Image–pro Plus 6.0 software (Media Cybernetics, United States), five visual fields were randomly selected in each slice of five heart tissue sections from each group.

#### Western Blotting

 According to the standard scheme, the expression of Bax and Bcl-2 in myocardial tissue was detected by Western blotting. Protein was extracted from myocardial tissue in a buffer containing RIPA lysate and protease inhibitor. The concentration of the protein was tested by BCA kit. The same amount of protein (30 μg) was transferred from SDS-polyacrylamide gel to polyvinylidene fluoride membrane. PVDF membrane was sealed for 1 h with 5% skim milk powder and incubated with primary antibody anti-Bax (AF0120, 1:2,000 dilution, Affinity, USA), anti-Bcl-2 (AF6139, 1:1,000 dilution, Affinity, United States), or anti-GAPDH (AB0036, 1:5,000 dilution, Abways, Shanghai, China) overnight at 4°C. PVDF membrane was washed 3 times with TBST for 10 min each time and incubated the second antibody labeled with HRP and shaked the bed for 1 h. The protein signal were detected by using enhanced chemiluminescence reagents (ECL) in Chemilluminescence Imaging System (ChemiScope 6,000 system pro, clinx, Shanghai, China) and analyzed by Quantity One software (Bio-Rad, Richmond, California, CA, United States).

#### Statistical Analysis

 Data are displayed as mean ± standard error of mean (SEM). Comparisons of the experimental groups were analyzed by one–way ANOVA with Bonferroni correction for post hoc test. *p* < 0.05 was considered as the indication of statistical significance. Data were analyzed with GraphPad Prism software (Version6.0, California, CA, United States).

## Results

### Characterization of aFGF-Nanoparticles (aFGF-NP) and Cationic Lipid Microbubbles

 The characteristic results of aFGF nanoparticles observed by transmission electron microscope were as follows, the nanoparticles were spherical, uniform size distribution and no adhesion. The average particle size of aFGF-NP measured by DLS method was 117.79 ± 1.63 nm. The Zeta potential was-11.32 mV. The entrapment efficiency of aFGF-NP was (84.73 ± 3.05)%.

**FIGURE 1:**
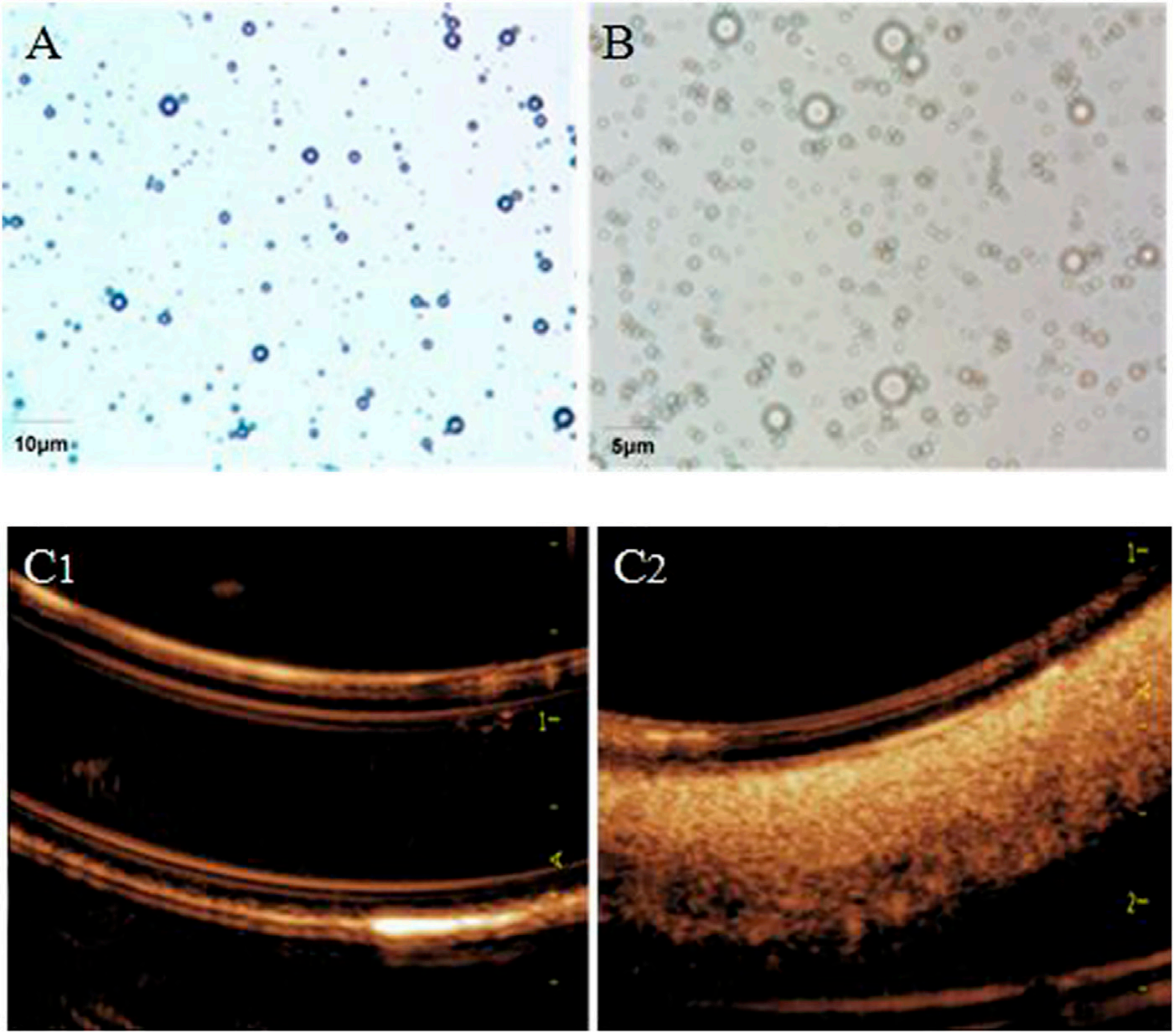
Light microscope view of cationic lipid microbubbles **(A)** and aFGF–NP + CPMBs **(B)**. Effect of aFGF–NP + CPMBs contrast–enhanced ultrasound *in vitro* (C1) and (C2), Comparison of echocardiography before and after aFGF–NP + CPMBs administration. aFGF, acidic fibroblast growth factor; NP, nanoparticles; CPMBs, cationic lipid microbubbles. Improvement of cardiac function dysfunction in DOX–HF.

The shape of CPMBs were regular, the surface was smooth and the size was basically uniform (Figure 1A). The average particle size was 4.27 ± 1.92 μm, and the average surface potential was +19.37 mV.

Carrying aFGF nanoparticles–cationic lipid microbubble complex (aFGF-NP + CPMBs): after mixing CPMBs with carrying aFGF heparinized nanoparticles (aFGF-NP), aFGF-NP could be successfully adsorbed on the surface of microbubbles because of the attraction of positive and negative charges on their surface, that was, aFGF nanoparticles–cationic lipid microbubble complexes (aFGF-NP + CPMBs) were formed (Figure 1B). The average particle size of the aFGF-NP + CPMBs was 4.39 ± 2.14 μm. There were no significant change in the appearance and size of microbubbles. It showed that CPMBs still had a good appearance after adsorbing liposomes. Absorbed aFGF-NP + CPMBs 100 μL, diluted it with normal saline 10 times and then put it in a rubber tube for imaging. The ultrasound mechanical index was 0.06 and the sound power was 20%. The microbubble showed obvious ultrasonic signal, indicating that the microbubble had a good contrast–enhanced ultrasound effect, laying the foundation for the following targeted therapy with drug delivery via microbubbles (Figures 1C1,2).

**FIGURE 2:**
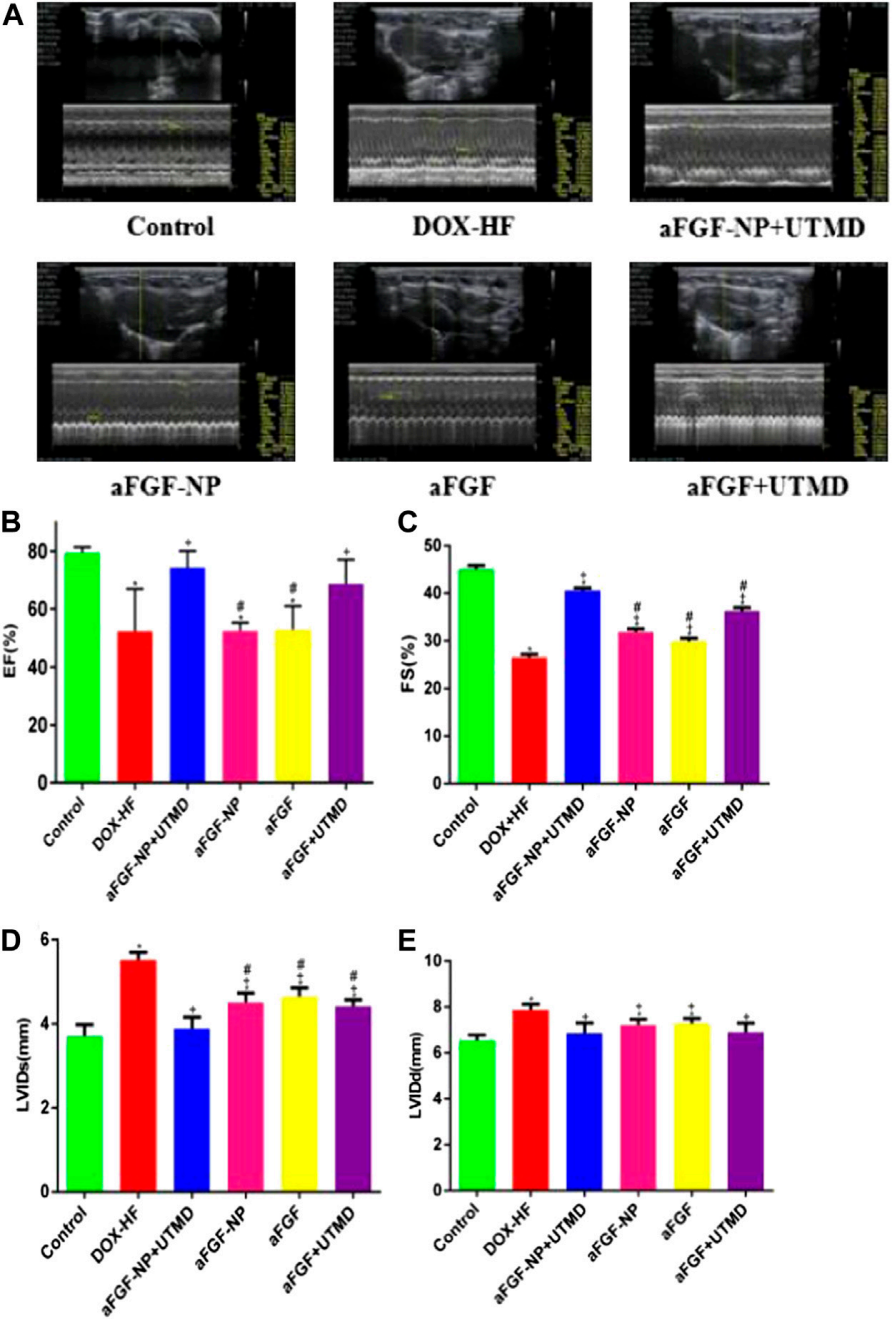
The aFGF–NP + CPMBs combined with UTMD prevented left ventricular dysfunction in DOX–HF rats. **(A)** Representative M–mode echocardiographic pictures of left ventricular function; **(B–E)** Quantitative analysis of EF(%), FS(%), LVIDs and LVIDd in each group. All data are expressed as mean ± SEM. *p < 0.05 vs. Control group; +*p* < 0.05 vs. DOX–HF group; #*p* < 0.05 vs aFGF–NP + UTMD group. aFGF, acidic fibroblast growth factor; NP, nanoparticles; CPMBs, cationic lipid microbubbles; UTMD, ultrasound targeted microbubble destruction; DOX, doxorubicin; HF, heart failure; EF, ejection fraction; FS, fractional shortening; LVIDs, Left ventricular internal diameter–systole; LVIDd, Left ventricular internal diameter-diastole. Reduction the level of myocardial injury and oxidative stress in DOX-HF.

M-mode echocardiography was used to detect the left ventricular function (Figures 2A–E). The ejection fraction (EF) and fractional shortening (FS) in DOX-HF group were decreased (*p* < 0.05) compared with the control group, which was increased by aFGF-NP + UTMD (*p* < 0.05) and aFGF + UTMD (*p* < 0.05) administration. Compared with the DOX-HF group, aFGF-NP group and aFGF group showed no significant difference in EF, but significant difference in FS (*p* < 0.05). Compared with the normal group, the LVIDs and LVIDd in the DOX-HF group increased (*p* < 0.05), but decreased in each group after treatment with aFGF (*p* < 0.05). Moreover, the echocardiographic results displayed that the therapeutic effect of aFGF-NP + UTMD group was better than that of aFGF + UTMD group in terms of improving left ventricular systolic function.

**FIGURE 3:**
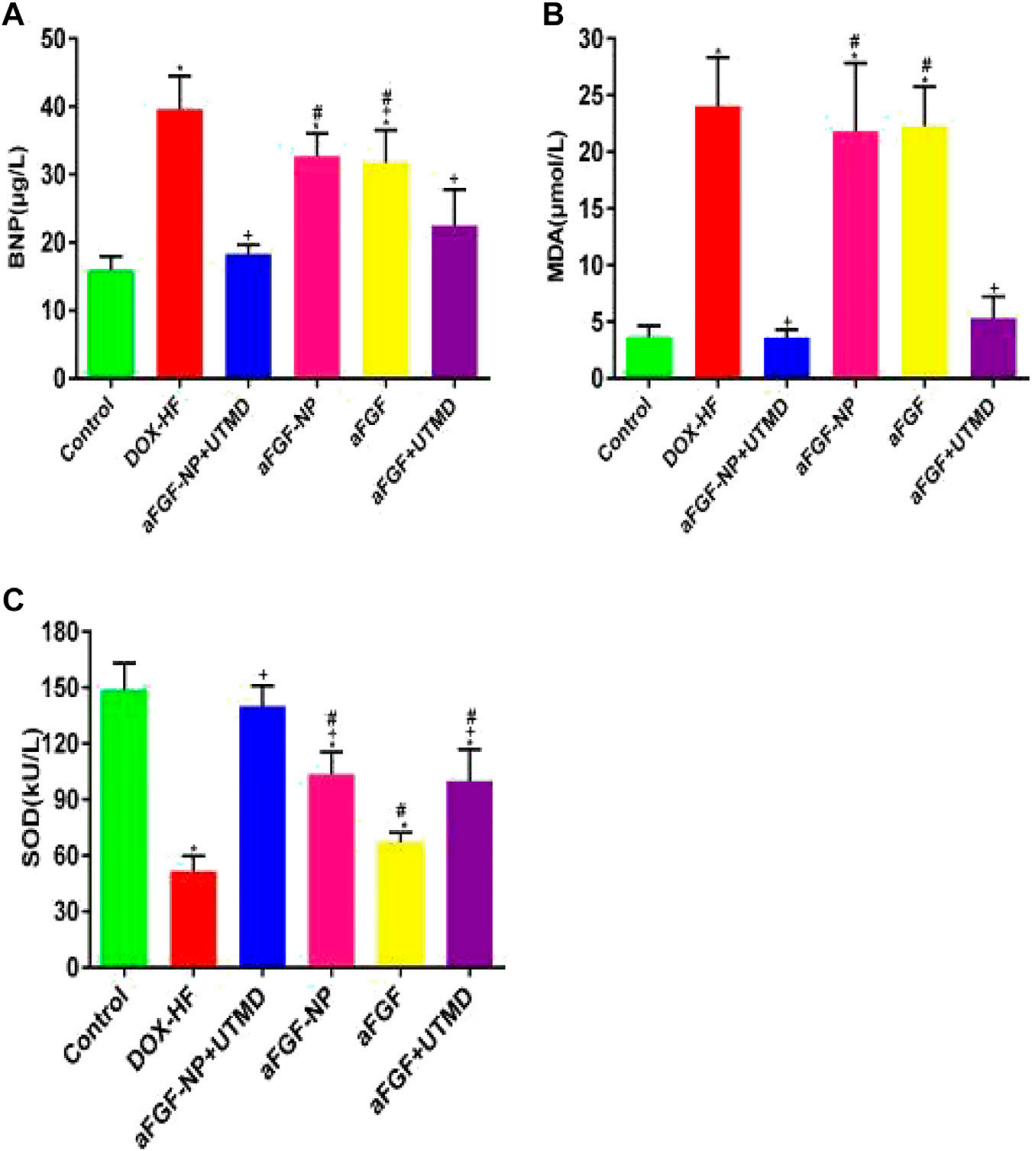
The levels of BNP in the serum **(A)**, MDA and SOD in myocardial tissue **(B, C)** were shown in each group. All data are expressed as mean ± SEM. *p < 0.05 vs. Control group; +*p* < 0.05 vs. DOX–HF group; #*p* < 0.05 vs. aFGF-NP + UTMD group. aFGF, acidic fibroblast growth factor; NP, nanoparticles; UTMD, ultrasound targeted microbubble destruction; DOX, doxorubicin; HF, heart failure; BNP, brain natriuretic peptide; MDA, malondialdehyde; SOD: superoxide dismutase. Improvement of myocardial histopathology in DOX–HF.

Doxorubicin-induced heart failure compared with the control group by evaluation of BNP (*p* < 0.05) (Figure 3A). The level of BNP in the other treatment groups was significantly lower than that in the DOX-HF group (*p* < 0.05, except for the aFGF-NP group, Figure 3A). Moreover, MDA and SOD were also detected (Figures 3B–C); MDA was increased obviously (*p* < 0.05) and SOD was decreased in the DOX-HF group apparently compared with the control group (*p* < 0.05); aFGF-NP + UTMD group (*p* < 0.05) and aFGF + UTMD group (*p* < 0.05) significantly lowered MDA levels and increased SOD levels compared with the DOX-HF group. Statistical results indicated that there was no difference in the level of MDA among the aFGF-NP group and aFGF group and the DOX-HF group. The level of SOD in the therapeutic groups was significantly higher than that in the DOX-HF group (*p* < 0.05, except for aFGF group). Moreover, the co–administration of aFGF-NP + UTMD demonstrated the lowest level of MDA and the highest level of SOD among all the therapeutic group, which noted that the foremost antioxidation effect was examined in the aFGF-NP + UTMD group.

**FIGURE 4:**
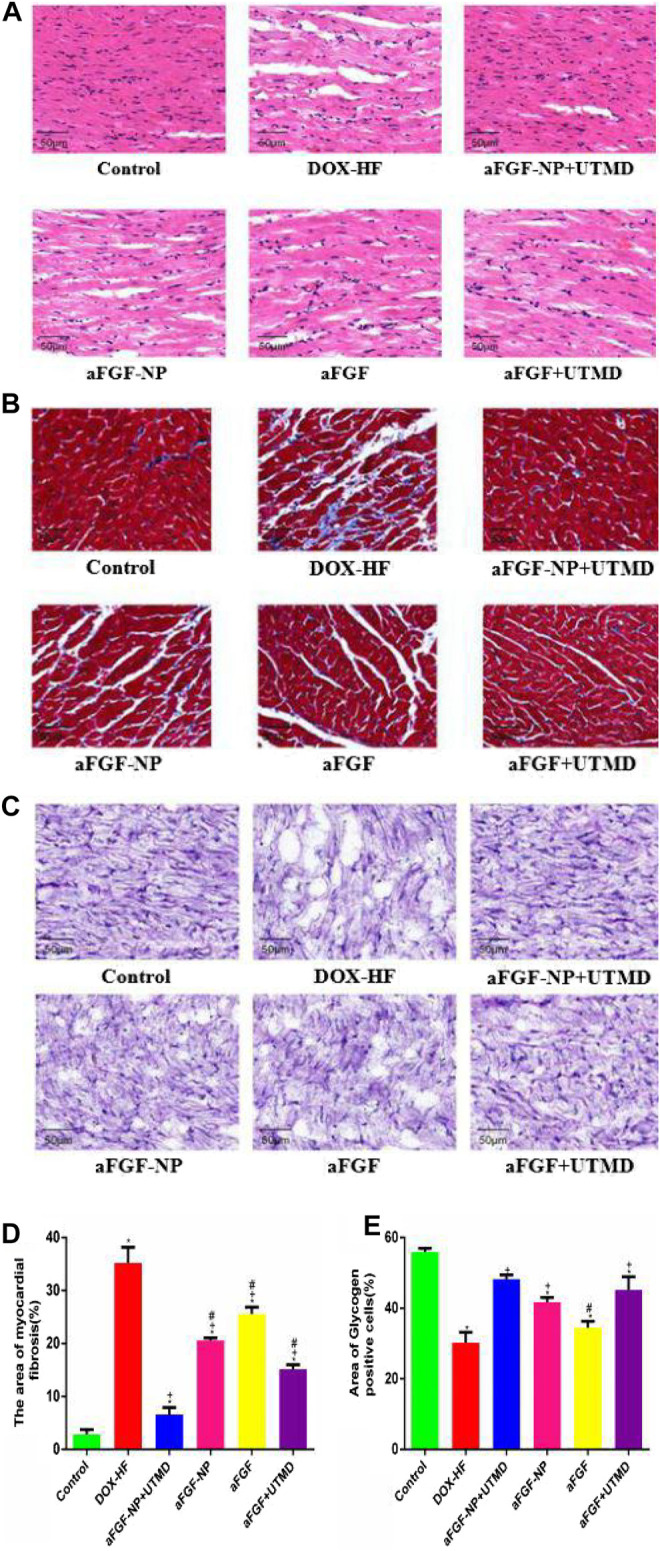
The aFGF–NP + CPMBs combined with UTMD alleviated the pathological changes of myocardial tissue in DOX–HF rats. **(A)**: Representative photos of HE staining (400x); **(B)**): Representative photos of Masson staining (400x); **(C)**: Representative photos of PAS staining (400x); **(D)**: Quantitative analysis of the degree of myocardial fibrosis in each group; **(E)**: Quantitative analysis of glycogen content in myocardial tissue of each group. All data are expressed as mean ± SEM. *p < 0.05 vs. Control group; +*p* < 0.05 vs. DOX–HF group; #*p* < 0.05 v.s aFGF–NP + UTMD group. aFGF, acidic fibroblast growth factor; NP, nanoparticles; CPMBs, cationic lipid microbubbles; UTMD, ultrasound targeted microbubble destruction; DOX, doxorubicin; HF, heart failure. Attenuation the apoptosis of myocardial tissue in DOX–HF.

The results of HE staining (Figure 4A)showed that compared with the control group, the DOX-HF group showed the disordered arrangement of cardiomyocytes, infiltration of inflammatory cells and focal vacuolation in the cytoplasm. Compared with the DOX-HF group, aFGF-NP, and aFGF showed the irregular arrangement of cardiomyocytes, interstitial edema and moderate inflammation. The pathological changes in the aFGF-NP + UTMD group were alleviated most significantly than those in other treatment groups. The results of Masson trichrome staining (Figures 4B–D) showed that the degree of fibrosis and myofibril degeneration and rupture in the DOX-HF group were significantly higher than those in the control group (*p* < 0.05). Compared with the DOX-HF group, the reduction of myocardial fibrosis was found in aFGF-NP and aFGF groups (*p* < 0.05). Quantitative analysis of myocardial fibrosis (Figure 4D) showed that aFGF-NP + UTMD could significantly reduce the degree of myocardial fibrosis in doxorubicin–induced heart failure compared with other treatment groups (*p* < 0.05). The results of glycogen PAS staining (Figures 4C–E) showed that the glycogen content of the DOX-HF group was significantly lower than that of the control group (*p* < 0.05). Compared with the DOX-HF group, glycogen content increased in aFGF-NP and aFGF + UTMD (*p* < 0.05), the quantitative analysis of glycogen content (Figure 4E) showed that aFGF-NP + UTMD could significantly improve the disturbance of glucose metabolism in doxorubicin-induced heart failure (*p* < 0.05).

**FIGURE 5:**
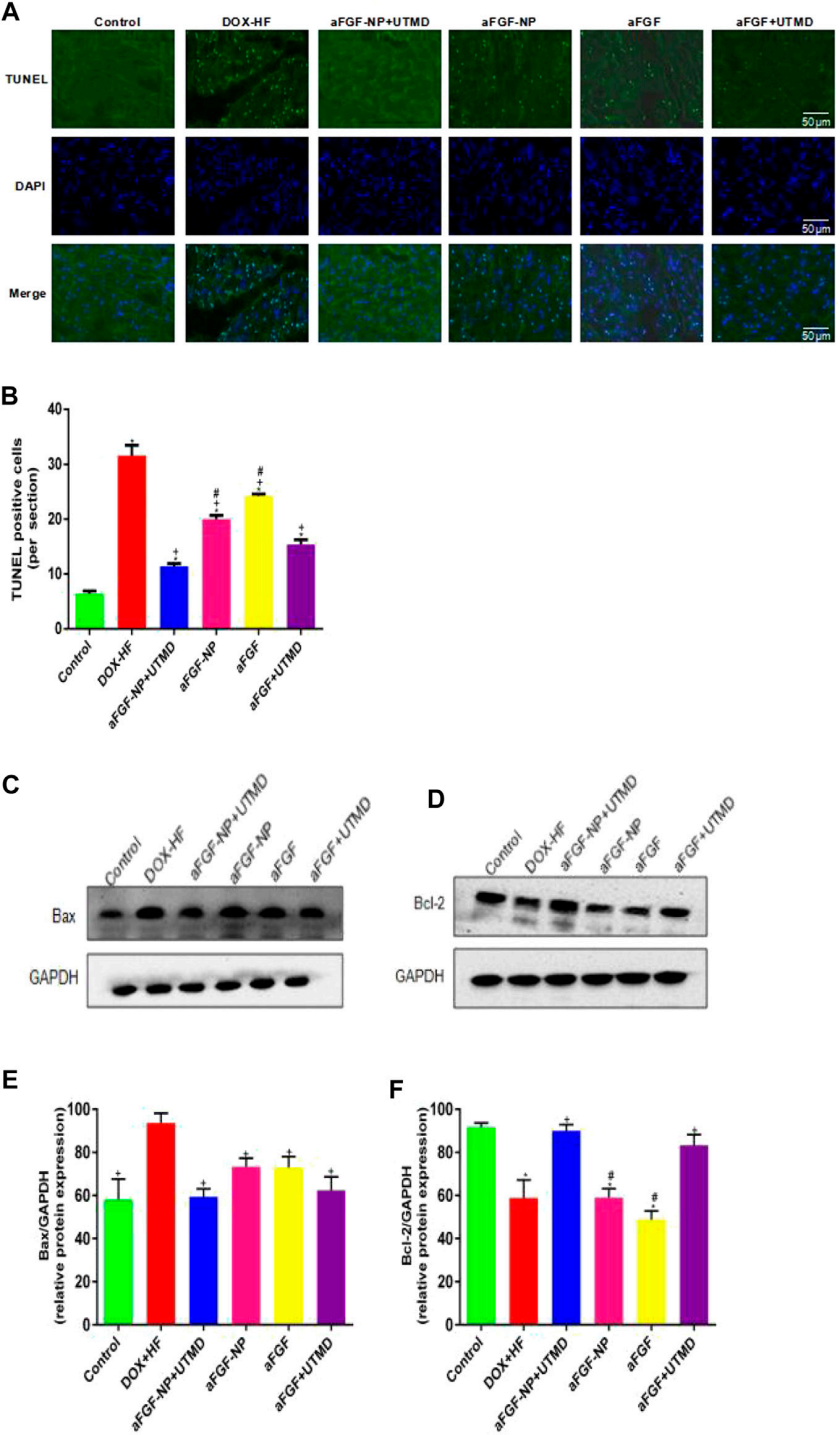
The aFGF–NP + CPMBs combined with UTMD inhibited cardiomyocyte apoptosis in DOX–HF rats. **(A)**: Representative photos of TUNEL staining in each group(400x); **(B)**: Quantitative analysis of the apoptotic cells. The protein expression of Bax and Bcl–2 in myocardial tissue was detected by Western blot. **(C, D)** Representative photos of Western blot; **(E, F)** Statistical analysis of the protein expression of Bax and Bcl–2. All data are expressed as mean ± SEM. *p < 0.05 vs. Control group; +*p* < 0.05 vs. DOX–HF group; #*p* < 0.05 vs. aFGF–NP + UTMD group. aFGF, acidic fibroblast growth factor; NP, nanoparticles; CPMBs, cationic lipid microbubbles; UTMD, ultrasound targeted microbubble destruction; DOX, doxorubicin; HF, heart failure. Prevention the decreation of myocardial capillary density in DOX–HF.

The effect of aFGF-NP + UTMD on cardiomyocyte apoptosis was performed by TUNEL staining (Figures 5A, B). There were very few TUNEL positive cells in the control group, but significantly increased in the DOX-HF group (*p* < 0.05) suggesting that cardiomyocytes were significantly apoptotic, while TUNEL positive cells decreased significantly after aFGF-NP + UTMD treatment (*p* < 0.05). The protein expression levels of Bax and Bcl-2 were analyzed by Western blot (Figures 5C–F), the expression level of Bax protein was higher and that of Bcl-2 protein was lower in the DOX-HF group, indicating an increase in cardiomyocyte apoptosis. On the contrary, compared with the DOX-HF group, the expression of Bax was significantly decreased and the expression of Bcl-2 was significantly increased in the aFGF-NP + UTMD group (*p* < 0.05), suggesting that cardiomyocyte apoptosis was reduced. Among the aFGF treatment groups, there was less apoptosis in the aFGF-NP + UTMD group compared with the other three groups.

**FIGURE 6:**
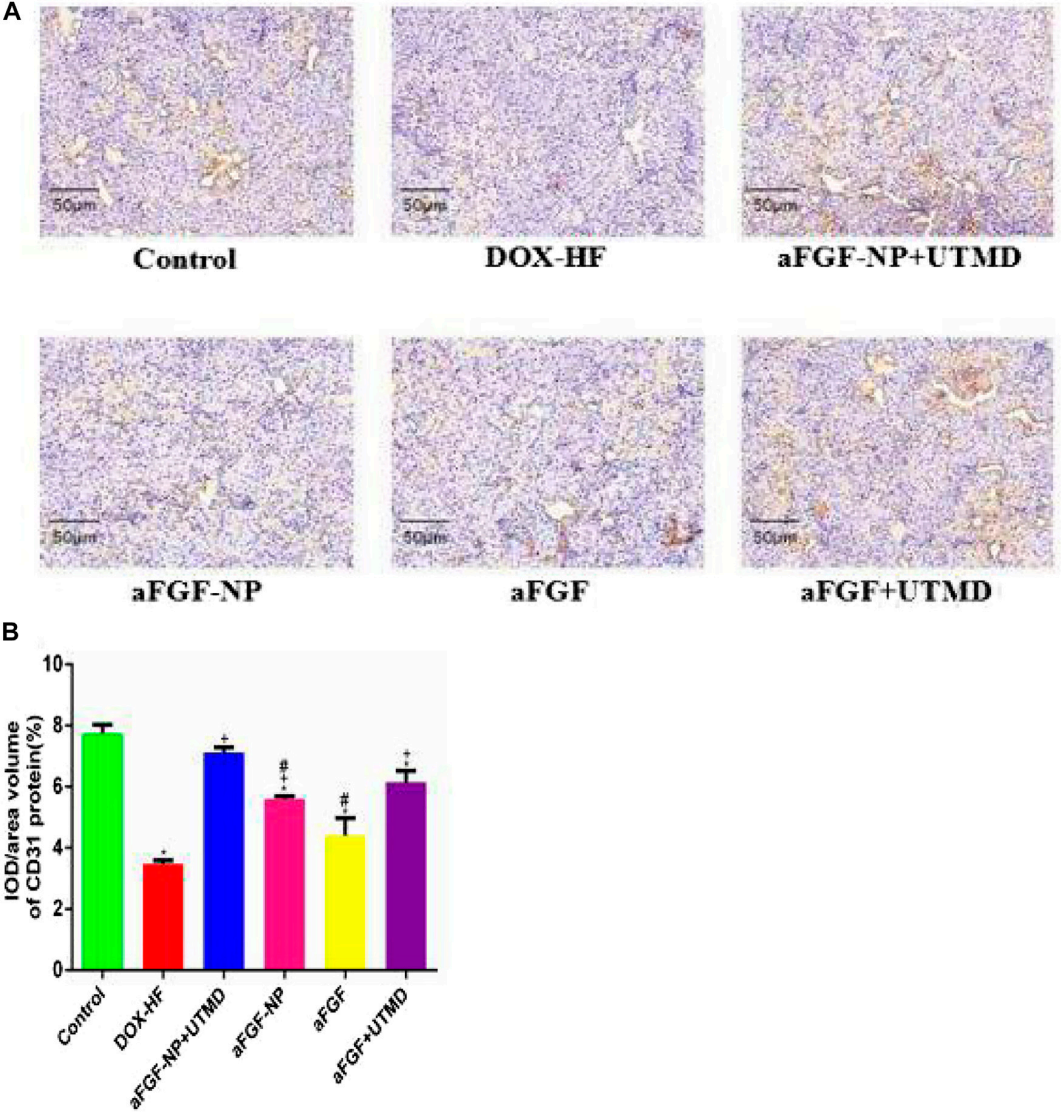
The aFGF–NP + CPMBs combined with UTMD promoted angiogenesis of myocardial tissue in DOX–HF rats. **(A)**.: Representative photos of CD31 immunohistochemical staining in each group (400x); **(B)**: Quantitative analysis of positive expression of CD31. All data are expressed as mean ± SEM. *p < 0.05 vs. Control group; +*p* < 0.05 vs. DOX–HF group; #*p* < 0.05 vs. aFGF–NP + UTMD group. aFGF, acidic fibroblast growth factor; NP, nanoparticles; CPMBs, cationic lipid microbubbles; UTMD, ultrasound targeted microbubble destruction; DOX, doxorubicin; HF, heart failure.

The role of aFGF-NP + CPMBs combined with UTMD technique in maintaining myocardial microvessels in DOX-induced heart failure was evaluated by immunohistochemical detection of myocardial angiogenesis marker CD31 (Figures 6A, B). The positive expression of CD31 was counted in 1 section which was divided into five regions. Compared with the normal group, the positive expression of CD31 in the DOX-HF group decreased significantly (*p* < 0.05); on the contrary, the treatment of aFGF-NP + UTMD could significantly prevent the decrease of positive expression of CD31 compared with the DOX-HF group (*p* < 0.05).

## Discussion

 Our research results have demonstrated the effect of aFGF targeted mediated by novel nanoparticles-cationic lipid microbubbles combined with UTMD in the therapy of heart failure as follows: 1) it can attenuate doxorubicin-induced heart failure and improve cardiac dysfunction; 2) it increases the activity of superoxide dismutase and decreases the formation of malondialdehyde, also down-regulates the expression of Bax and up-regulates the expression of Bcl-2 in cardiac myocyte, thus inhibits cardiomyocyte oxidative stress and apoptosis in heart failure; 3) it promotes angiogenesis in cardiac tissue during heart failure via increasing the expression of CD31; proposing a novel mechanism for anti-heart failure cardioprotection of aFGF targeted mediated by novel nanoparticles-cationic lipid microbubbles combined with UTMD.

The main pathological features of heart failure are concentric or eccentric myocardial hypertrophy, decreation of cardiomyocytes caused by necrosis or apoptosis, myocardial fibrosis, disturbance of energy production, and reduction of capillary density in hypertrophic myocardium (Goldenthal, 2016).The above pathological changes lead to myocardial remodeling, which further leads to the decrease of myocardial compliance, that is, the increase of stiffness, which affects the systolic and diastolic function of the myocardium (Ruan et al., 2018). Considering the high incidence of heart failure in acute myocardial infarction, dilated cardiomyopathy and other cardiovascular diseases, especially in the elderly, the cardiomyocyte apoptosis and the reduction of capillary density is closely related to the pathogenesis of heart failure (Moe and Marín-García, 2016). Nevertheless, owing to the finite acquaintances of the latent mechanisms, there are few effective therapeutic methods to prevent cardiomyocyte apoptosis and decreation of capillary density in the treatment of heart failure.

Acidic fibroblast growth factor (aFGF) is a key member of the fibroblast growth factor (FGF) family. Compared with basic fibroblast growth factor (bFGF), aFGF has a strong affinity for heparin and myocardial tissue. AFGF can promote the proliferation of vascular endothelial cells and smooth muscle cells, thus promote angiogenesis, alleviate myocardial ischemia and improve cardiac function (Zhang et al., 2016). Zhao YZ.et al. reported that the aFGF combined with heparin modified microbubbles under UTMD-mediated cavitation to achieve the cardiac protection for acute myocardial infarction (Zhao et al., 2012). Fan CM.et al. found that aFGF could benefit the repair of the myocardium and the improvement of cardiac function after myocardial infarction in mice by reducing infarct size and promoting angiogenesis (Fan et al., 2020). Zhang C.et al. confirmed that non-Mitogenic aFGF could prevent diabetic cardiomyopathy from cardiac oxidative stress damage, cardiomyocyte hypertrophy and myocardial fibrosis (Zhang et al., 2013). Zhang M.et al. also suggested that heparin-modified microbubbles carrying aFGF combined with the UTMD technique could inhibit abnormalities such as left ventricular dysfunction, myocardial fibrosis, cardiomyocyte apoptosis and microvascular sparse (Zhang et al., 2016). Therefore, aFGF is a potentially valuable drug for the treatment of heart failure.

For the sake of strengthening the stability of the drug, aFGF-NP was prepared by water-in-water emulsification combined with freeze-drying technology. Through the analysis of its physical and chemical properties, it was demonstrated that the drug-loaded nanoparticles were spherical, small particle size, even dispersion. It had a certain Zeta potential and could maintain good stability. The temperature of the whole preparation process was controlled below 20°C, which had the advantages of a short time and high efficiency, which prevented the protein drugs from being degraded as far as possible.

Echocardiography is a non-invasive and multi-functional tool for collecting images and data of heart structure and function. Brain natriuretic peptide (BNP), a natural hormone with biological activity synthesized by cardiomyocytes, is mainly expressed in the ventricle. When the left ventricle is dysfunctional, it is rapidly synthesized and released into the blood due to myocardial dilation. As a sensitive marker of heart failure, BNP reflects not only left ventricular systolic and diastolic dysfunction, but also valvular dysfunction and right ventricular dysfunction (Li et al., 2019). In accordance with previous results, EF and FS decreased significantly, while LVIDs, LVIDd and BNP increased abnormally in the doxorubicin-induced heart failure animal model group, (Hang et al., 2017; Wen et al., 2019) indicating that there was serious cardiac damage and left ventricular dilatation, that is, the cardiac pumping function decreased. We found that the aFGF-NP + CPMBs combined with UTMD could increase EF and FS, as well as decrease LVIDs, LVIDd, and BNP, compared with the DOX-HF group, which proved to prevent the deterioration of cardiac function induced by doxorubicin.

The results of cardiac histopathology were consistent with other studies, such as hypertrophy and disorder of cardiomyocytes, infiltration of inflammatory cells, disorder and rupture of myocardial fiber structure, and increase of fibrosis degree (Ma et al., 2017; Sheibani et al., 2020). These pathological changes of heart failure were obviously mitigated by aFGF-NP + CPMBs combined with UTMD. Interestingly, through the PAS staining of glycogen in myocardial tissue, we had a surprising discovery that aFGF-NP + CPMBs combined with UTMD group compared with heart failure model group, muscle glycogen decomposition was reduced. During heart failure due to hypoxia, which resulted in the increase of intracellular phosphofructokinase activity, the body mobilizes muscle glycogen to decompose and produce ATP through glycolysis to supply energy for heart contraction; while the enhancement of glycolysis activity will lead to energy deficiency and lactic acid accumulation, which will eventually worsen HF. This finding suggested that the protective effect of aFGF-NP + CPMBs combined with UTMD on heart failure may be related to reducing muscle glycogen decomposition and glycolysis, promoting tricarboxylic acid cycle and improving myocardial energy metabolism, which needs to be further studied. This result was similar to the cardioprotective effect of Qishen granule that regulated fatty acid and glucose metabolism to improve myocardial energy metabolism in rats with chronic heart failure (Gao et al., 2020).

The pathophysiological mechanism of heart failure caused by doxorubicin involves a variety of reasons, including oxidative stress, cardiomyocyte apoptosis, inflammation, calcium overload, iron homeostasis and mitochondrial dysfunction. Among them, oxidative stress leads to damage of the lipid membrane and other cellular components due to the imbalance between the oxidant/antioxidant enzyme system, which is the main cause of heart failure after the cumulative dose of doxorubicin. Doxorubicin is an anthracycline-containing quinone, which binds to cardiolipin in the mitochondria of cardiomyocytes. Its quinone part produces reactive oxygen species (ROS) through redox reaction in mitochondrial electron transport chain complex1 (Mohammed et al., 2020; Sheibani et al., 2020). Doxorubicin could combine with iron to form Fe3^+^ doxorubicin complex, which led to the deactivation of cytochrome c oxidase and changed the iron homeostasis process related to aconitase-ferritin-1. Cytochrome c is an important part of the electron transport chain, and the reduction of its activity mediates the production of free radicals (Mohammed et al., 2020).

The heart lacks antioxidant enzymes to scavenge free radicals. At the same time (Zare et al., 2019) the accumulation of free radicals causes lipid peroxidation, damages cell and mitochondrial membrane, endoplasmic reticulum and intracellular macromolecules (Sheibani et al., 2020) destroys the structure and function of normal cardiomyocytes, and leads to cardiac dysfunction, and participates in the progress of HF (Qi et al., 2020). Superoxide dismutase (SOD) is a kind of antioxidant enzyme with the function of scavenging free radicals and protecting cells from damage (Zare et al., 2019). Malondialdehyde (MDA) is a marker of lipid peroxidation. Compared with the control group, SOD decreased and MDA increased in the heart failure model group. It is confirmed that doxorubicin can cause damage to cardiomyocytes by enhancing oxidative stress. This result is in line with many previous reports. Compared with the heart failure model group, aFGF-NP + CPMBs combined with UTMD treatment group significantly increased SOD and decreased MDA in myocardial tissue. The results showed that aFGF-NP + CPMBs combined with the UTMD technique could protect cardiomyocytes from oxidative stress injury induced by doxorubicin.

Doxorubicin induces oxidative stress, resulting in the production of a great deal of ROS (Sheibani et al., 2020) which changes the permeability of mitochondria (Kaludercic and Di Lisa, 2020). The formation of disulfide bonds around the mitochondrial permeability transition pore (MPTP) or MPT leads to the opening of the mitochondrial MPT pore (Sabet et al., 2020) and ROS can induce the opening of the MPT pore (Kaludercic and Di Lisa, 2020). Once opened, the metabolites and ions in the MPT pore dissipate grads, proton power collapses, resulting in the rupture of the outer membrane of the mitochondria and the swelling of a large number of mitochondria (Sabet et al., 2020). The opening of MPT leads to the release of apoptosis factors (such as cytochrome C)from the mitochondria to the cytoplasm and initiates the endogenous mitochondrial apoptosis pathway (Morciano et al., 2015). As known apoptosis regulators, the Bcl-2 family plays a critical role in apoptosis induced by various stimuli (ischemia, hypoxia, inflammation and oxygen free radicals, etc.). Apoptosis is directly regulated by the protein level of Bcl-2 and Bax protein. The promoter and executor of mammalian cell apoptosis are the Caspases, Caspases cascade downstream of the most critical apoptotic protease is cleaved Caspase-3. The increase of Bcl-2 inhibits apoptosis by interfering with the release of cytochrome C to block the activation of Caspase-3. The increase of Bax promotes cytochrome C to pass through the mitochondrial membrane, activate Caspase-9, and then activate Caspase-3, which leads to apoptosis (Zhu et al., 2018). Cytochrome C-mediated apoptosis plays a key role in cardiomyocyte apoptosis. Mitochondrial dysfunction and cardiomyocyte apoptosis are closely related to the follow-up development of heart failure. Consistent with the results of previous research (Ammar et al., 2011) we found that the expression level of Bcl-2 protein decreased significantly and the expression level of Bax protein increased significantly in the myocardial tissue of doxorubicin-induced heart failure animal model group, suggesting that cardiomyocyte apoptosis plays an important role in the progression of heart failure. After the intervention of aFGF-NP + CPMBs combined with UTMD, the expression of Bcl-2 protein increased significantly, while the expression of Bax protein decreased significantly, suggesting that the cardiac-protective effect of aFGF-NP + CPMBs combined with UTMD on doxorubicin-induced heart failure is related to its anti-apoptosis characteristics.

The cardiac pumping function decreases in failing heart, which results in the activation of renin-angiotensin-aldosterone system (RAAS). RAAS is an important compensatory mechanism in cardiac dysfunction, which increases the level of plasma angiotensin Ⅱ (AngⅡ). AngⅡ not only promotes cardiomyocyte hypertrophy and fibroblast proliferation, but also induces angiogenesis damage secondary to myocardial hypertrophy. Recent studies have shown that AngⅡ-induced angiogenesis damage was closely related to p53-dependent downregulation of HIF-1 mediated by Jagged1/Notch1 signal pathway (Guan et al., 2017). The decrease of capillary density in hypertrophic myocardium and the increase of diffusion distance from capillaries to the center of cardiomyocytes lead to myocardial ischemia and hypoxia, which is an important mechanism leading to heart failure (Ruan et al., 2018; Sui et al., 2019).

The fibroblast growth factor is a recognized mitogen, which plays an important role in the differentiation, proliferation, metastasis and chemotaxis of vascular endothelial cells by binding to specific receptors expressed on the cell surface (Chen et al., 2020). CD31, also known as the platelet-endothelial cell adhesion molecule, with a molecular weight of 130kDa, belongs to the immunoglobulin superfamily. It exists in the tight junction between endothelial cells and participates in angiogenesis. The decreation of capillary density was observed in the myocardium of heart failure induced by doxorubicin (Ammar et al., 2011) which was determined by the reduction of angiogenesis symbol CD31, which was consistent with our study. Our study confirmed that after aFGF-NP + CPMBs combined with UTMD treatment, the expression of CD31 increased, which increased the blood vessel density in ischemic myocardium. This is the first time we have reported that aFGF-NP + CPMBs combined with UTMD alleviated the symptoms of heart failure by promoting angiogenesis and improving the perfusion of the ischemic myocardium. However, the mechanism of aFGF-NP + CPMBs combined with UTMD promoting angiogenesis needs to be further studied. In addition, it was also confirmed that growth factor aFGF was significantly up-regulated during the growth of canine cardiac collateral vessels, suggesting that the production of endogenous growth factors may be an important factor in promoting the growth of collateral vessels (Guan et al., 2016). In the skin wound model of C57BL/6 mice, treating the wound with HP-aFGF hydrogel can increase the expression of CD31 and promote angiogenesis to lead to rapid wound healing (Wu et al., 2016).

**FIGURE 7:**
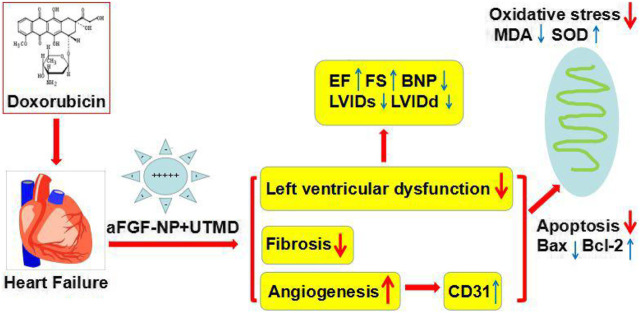
The therapeutic mechanism of the aFGF targeted mediated by novel nanoparticles microbubble complex combined with UTMD inhibition of myocardial oxidative stress, fibrosis, apoptosis, and promotion of cardiac angiogenesis in doxorubicin induced–heart failure rats.

To sum up, this research offered evidence *in vivo*, which intensely suggested that the combined use of aFGF targeted mediated by novel nanoparticles-microbubble complex combined with UTMD could effectively antagonize cardiac damage induced by DOX and protect left ventricular cardiac function in DOX-HF rats, and its mechanism may be related to anti-oxidation, anti-fibrosis, anti-apoptosis and cardiac angiogenesis (Figure 7). As a result, this research provides a bright future for the treatment of heart failure induced by DOX in the clinic.

## Data Availability

The raw data supporting the conclusions of this article will be made available by the authors, without undue reservation.
